# Synthesis of Vertically Aligned Porous Silica Thin Films Functionalized by Silver Ions

**DOI:** 10.3390/ijms22147505

**Published:** 2021-07-13

**Authors:** Andrii Fedorchuk, Alain Walcarius, Magdalena Laskowska, Neus Vila, Paweł Kowalczyk, Krzysztof Cpałka, Łukasz Laskowski

**Affiliations:** 1 Institute of Nuclear Physics, Polish Academy of Sciences, 31-342 Krakow, Poland; andrii.fedorchuk@ifj.edu.pl (A.F.); lukasz.laskowski@ifj.edu.pl (Ł.L.); 2 CNRS, LCPME, Université de Lorraine, 54000 Nancy, France; alain.walcarius@univ-lorraine.fr (A.W.); neus.vila@univ-lorraine.fr (N.V.); 3 Department of Animal Nutrition, The Kielanowski Institute of Animal Physiology and Nutrition, Polish Academy of Sciences, 05-110 Jabłonna, Poland; p.kowalczyk@ifzz.pl; 4 Institute of Computational Intelligence, Czestochowa University of Technology, 42-200 Czestochowa, Poland; krzysztof.cpalka@pcz.pl

**Keywords:** mesoporous silica thin films, electro-assisted self-assembly, functional materials, electrochemistry

## Abstract

In this work, we have developed a chemical procedure enabling the preparation of highly ordered and vertically aligned mesoporous silica films containing selected contents of silver ions bonded inside the mesopore channels via anchoring propyl-carboxyl units. The procedure involves the electrochemically assisted self-assembly co-condensation of tetraethoxysilane and (3-cyanopropyl)triethoxysilane in the presence of cetyltrimethylammonium bromide as a surfactant, the subsequent hydrolysis of cyano groups into carboxylate ones, followed by their complexation with silver ions. The output materials have been electrochemically characterized with regard to the synthesis effectiveness in order to confirm and quantify the presence of the silver ions in the material. The mesostructure has been observed by transmission electron microscopy. We have pointed out that it is possible to finely tune the functionalization level by controlling the co-condensation procedure, notably the concentration of (3-cyanopropyl)triethoxysilane in the synthesis medium.

## 1. Introduction

Mesoporous silica thin films built of silicon dioxide (mainly amorphous) have an extraordinary developed surface due to the presence of a vast amount of pores in the structure [1], usually ordered [2]. Depending on the geometry of pores and their mutual location, these materials can possess no real ordering (have a worm-like structure) or can be 1D or 2D structured, 3D cage structured, or 3D continuous [3,4,5,6,7]. Moreover, each mentioned type of structure can be divided deeper into numerous sub-types. For example, 2D structured thin films can possess pores with various diameters, distributed irregularly [8,9] or regularly with possible hexagonal or rectangular arrangement [10,11,12], laying parallel [13,14] or perpendicular [15] to the surface. These latter nanometric layers can be mainly divided into two classes—deposited on various substrates or free standing [16,17,18,19].

**Figure 1:**
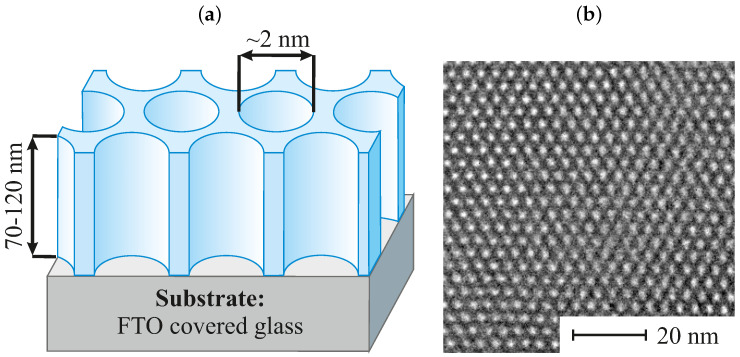
The structure of a vertically aligned porous silica thin film prepared via electro-assisted self-assembly method (EASA): scheme (**a**) and TEM microphotography (**b**).

From the applicative point of view, it is essential to adjust the nanostructure of mesoporous thin films in order to ensure good accessibility and fast transport properties [7,20,21,22]. In this respect, regular nanoporous thin films containing vertically aligned mesochannels [8,16,23] offer an ideal configuration for being used as matrices for the fabrication of functional materials with sophisticated structures accompanying unique properties. An efficient way to prepare them is the electro-assisted self-assembly method (EASA), resulting in uniform thin films made of vertically aligned mesopore channels, ordered in a 2D hexagonal pattern, with a pore diameter of about 2 nm and wall thickness of 1 nm [23,24]. The structure of pristine EASA thin films (non-functionalized) can be seen in Figure 1a along with the transmission electron microscopy (TEM) micrograph showing the perfect hexagonal arrangement of the packed mesopores (Figure 1b).

Despite their extraordinary structure, the real potential of porous silica thin films became clear after taking into account the possibility of their functionalization [4,25,26,27,28]. From this point of view vertically aligned mesoporous silica layer can be treated as a unique template that can be precisely modified for particular needs via the deposition of corresponding units (functional groups [29,30], molecules [31,32], nanoparticles [33,34], nanowires [35,36,37], etc. [38]). Thanks to the extraordinarily high specific surface area, such a matrix can accommodate a huge number of anchoring moieties on its internal pore surface and a large variety of organo-functional groups owing to the possible combination of EASA with click chemistry [39,40]. What is more, we are capable of finely tuning their number, realizing by it the idea of the “2D solid solvent” [41].

Such materials can find applications mainly as antimicrobial coatings. As we reported earlier [42], functionalized mesoporous silica can present very unusual biocidal properties. The rate of bacteria elimination strongly depended on the concentration of metal-containing molecules, but this dependency was not linear and not monotonous. In this case, the precise control of the doping rate was crucial for the material’s properties. Another application where the precise control of the distribution of the functional units is vital can be the fabrication of layouts of magnetic units. Such systems are essential for the vision of super-dense memory storages [43] or even molecular neurons [44]. In addition, in this case, the density and distribution of functional units play an essential role in the magnetic properties of the materials [45,46]. Looking at the examples mentioned above, the precise tuning of the number of functional units inside silica pores seems justified.

**Figure 2:**
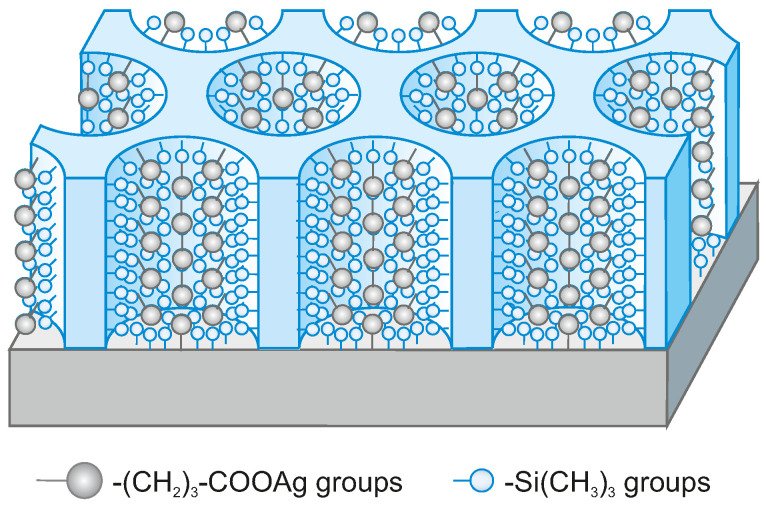
The scheme of a vertically aligned porous silica thin film containing silver ions anchored inside pores via propyl-carboxyl units.

This study presents an effective procedure allowing for the fabrication of the vertically aligned mesoporous silica thin films containing silver ions anchored inside the mesopores via propyl-carboxyl units. The structure of the assumed material can be seen in Figure 2. The number of the silver ions accommodated inside pores can be tuned by the variation in the number of anchoring propyl-carboxyl units incorporated in the silica structure during the co-condensation synthesis procedure. The materials developed here have great importance as far as antimicrobial layers are concerned. What is more, such compounds can be a starting point for the fabrication of the much more sophisticated nanostructured composite materials, containing for instance metallic nanowires inside pores (regularly 2D distributed).

## 2. Materials and Methods

### 2.1. Characterization Methods

The electrodeposition of silica thin films (chronoamperometry with controlled potential) as well as their analysis by differential pulse anodic stripping voltammetry (DPASV) was carried out using potentiostat/galvanostat SP150 (Biologic) at room temperature in the three-electrode configuration.

The working electrode was a conductive substrate (fluoride-doped tin oxide (FTO)-covered glass) in the case of electrodeposition of silica thin films (see next section) and the same substrate with deposited thin films containing the anchored silver ions for DPASV measurements. As the counter electrode, we used a platinum plate, while the reference electrode was standard Ag/AgCl. DPASV measurements were carried out in 0.1 M NaNO${}_{3}$ electrolyte solution. For synthesis and electrochemical experiments, we used custom fabricated Teflon cell, allowing for the application of the substrate at the bottom.

The TEM imaging was carried out using the FEI Tecnai G2 20 X-TWIN electron microscope, equipped with emission source LaB6 and CCD camera FEI Eagle 2K. The TEM images were processed (Fourier frequency transformation) using the Gwyddion software [47].

### 2.2. Synthesis of Vertically Aligned Mesoporous Silica Thin Films Functionalized with Silver Ions

**Figure 3:**
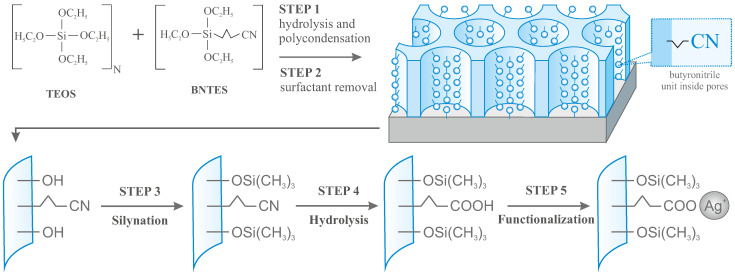
The general procedure of synthesis of vertically aligned mesoporous silica thin films with 2D hexagonally arranged pores containing silver ions inside pores with assumed concentration.

The preparation of vertically aligned mesoporous silica thin films with hexagonally packed mesochannels containing silver ions at selected and controllable content can be divided into the five steps summarized in Figure 3.

As appropriate substrates, we used glass plates covered pyrolytically by fluoride-doped tin oxide (FTO) conductive layer (the material with increased mechanical and chemical resistance with the surface resistivity = 6${}^{-10}$ Ω purchased from 3D Nano Ltd., Krakow, Poland). It is important to use the pyrolytically layered glass because only this procedure assures the FTO coating strong enough for the harsh reaction involving in the synthesis procedure. Solvents were dried out and distilled just before use. Reagents with the highest purity available were used for the reactions. NaNO${}_{3}$ was purchased from Chempur Ltd., Piekary Slaskie, Poland. AgNO${}_{3}$, Cetyltrimethylammonium bromide (CTAB), tetraethylorthosilicate (TEOS), trimethylsilyl chloride (TMSCl) and 4-(triethoxysilyl)butyronitrile (BNTES), also known as (3-cyanopropyl)triethoxysilane, were purchased from Merck Ltd., Darmstadt, Germany.

At first, the substrates were washed mechanically using a detergent. Next, they were washed a few times with deionized water in an ultrasonic bath, followed by washing with n-propanol. Finally, FTO glasses were soaked in a 1:1 solution of concentrated HCl and ethanol for 15 min and rinsed a few times with deionized water in the ultrasonic bath. Thoroughly cleaned substrates were dried under nitrogen flow and stored under a protective atmosphere.

The procedure of electro-assisted generation of functionalized silica thin films (**STEP 1**) starts with preparing the starting sol made of 20 mL aqueous solution containing 0.1 M NaNO${}_{3}$, 20 mL of ethanol, 0.47 g of CTAB, and 4 millimoles of the silica precursors mixture: TEOS and BNTES. The molar ratio between the two last compounds defines the doping level of the final materials (the N number in Figure 3 being the number of TEOS molecules used for one BNTES). In order to yield final molar concentrations of functional units of 10%, 5%, 2.5%, and 1.25% (SiO-R molecules in all silica-containing molecules), we assumed the following proportions of TEOS to BNTES: 9:1, 19:1, 39:1, and 79:1 (N numbers of 9, 19, 39, and 79). The mixtures were stirred for 15 min till CTAB was dissolved. Next, the pH was decreased to 3 by means of the addition of HCl (0.1 M in water). The sol solution was mixed for three hours to achieve hydrolysis of the precursors.

The surfactant-templated silica films were grown by applying a cathodic potential of +1.5 V for 20 s against the Ag/AgCl reference electrode after immersion of the FTO electrode in the hydrolyzed sol solution. The procedure resulted in 100 nm thick porous silica thin films. After film generation, the electrode surface was immediately rinsed with water and aged overnight at a fixed temperature of 130 °C to complete the polycondensation process and harden the newly obtained mesostructure.

To extract the surfactant template from the film (**STEP 2**), the samples were dipped into an ethanolic solution containing 0.1 M HCl and maintained under moderate stirring for 15 min. After washing a few times with ethanol, the samples were dried under a vacuum for a night.

The materials after this stage contain functional butyronitrile (3-cyanopropyl) groups inside open pores, although silanol groups (–OH) are also present at the pore surface (onto the internal silica walls). As these later molecules can react with a carbon atom at the end of butyronitrile groups during hydrolysis (further step), they are likely to make the target anchoring units incapable to bonding silver ions. For this reason, we passivated surface hydroxyl units by silanization (**STEP 3**). To this end, we treated the samples with 2% trimethylsilyl chloride in dichloromethane (CH${}_{2}$Cl${}_{2}$—Chempur Ltd., Piekary Slaskie, Poland). The reaction was carried out in the Teflon-Parr autoclave at the temperature of 70 °C for 24 h. After this time and cooling down, the samples were washed a few times with dichloromethane and dried under vacuum at a fixed temperature of 100 °C overnight.

As-prepared materials were hydrolyzed in the next step (**STEP 4**) in order to transform cyanopropyl group into propyl carboxylic acid units, capable of immobilization of silver ions. This was achieved from a solution containing concentrated hydrochloric acid (37%—Chempur Ltd.) in acetone and water (0.9:0.9:0.2 *v*:*v*:*v*). The addition of acetone is very important in this case because the interior of the pores is hydrophobic. As for the previous step, the reaction was also performed in this case in the Teflon-Parr autoclave at a temperature of 70 °C for 24 h. After cooling down, the samples were washed a few times with a mixture of water and acetone and dried overnight under vacuum at 100 °C.

At the final step (**STEP 5**), the prepared samples were activated with silver ions. This was achieved by contacting the pre-functionalized porous silica matrices containing carboxylic acid anchoring units to a solution of AgNO${}_{3}$ in hydro-organic medium 1 × 10${}^{-2}$ M AgNO${}_{3}$ in 1:1 mixture of water and acetone). The reaction proceeded at the temperature of 70 °C inside the Teflon-Parr autoclave. All experiments were carried out in total darkness in order to avoid any decomposition of the AgNO${}_{3}$ solution into metallic silver. After washing a few times with a mixture of water and acetone and drying, samples were ready to use. When not used, the obtained materials were stored in total darkness under a protective atmosphere of argon.

Here it is worth mentioning that the final concentration of silver inside silica channels is determined by the content of the propyl carboxylic acid units: single anchoring groups are capable of immobilizing a single silver ion, creating a silver carbonate molecule. Summing up, we obtained the following samples:

- vertically aligned porous silica thin films containing 10% of propyl-silver carbonate units inside pore—1 OSiR group per 9 SiO${}_{2}$—**SIL-prop-COOAg 9**;
- vertically aligned porous silica thin films containing 5% of propyl-silver carbonate units inside pore—1 OSiR group per 19 SiO${}_{2}$—**SIL-prop-COOAg 19**;
- vertically aligned porous silica thin films containing 2.5% of propyl-silver carbonate units inside pore—1 OSiR group per 39 SiO${}_{2}$—**SIL-prop-COOAg 39**;
- vertically aligned porous silica thin films containing 1.25% of propyl-silver carbonate units inside pore—1 OSiR group per 79 SiO${}_{2}$—**SIL-prop-COOAg 79**.

## 3. Results and Discussion

**Figure 4:**
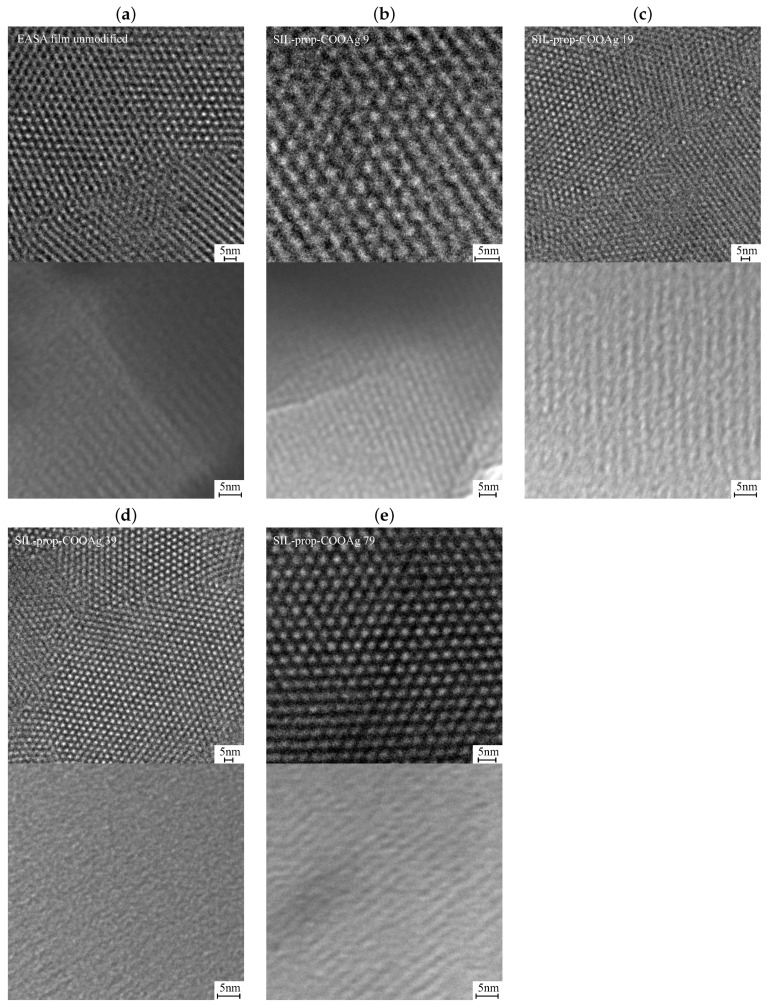
TEM images of the porous silica thin layers unmodified (**a**) and containing different concentrations of the functional groups (**b**–**e**). Figures show plan views (upper images) and cross-section views (bottom images).

The first evidence of the obtained materials’ correctness was done by means of TEM microscopy. As it can be seen in Figure 4, all the samples have the expected mesostructure made of hexagonally packed channels that grew perpendicular to the electrode substrate. The pores diameter is about 2 nm, while inter-planar distance is about 3.5 nm, consistent with earlier reports on the same kind of oriented films [23,24]. Interestingly, no differences between pure silica thin films and functionalized samples can be noticed, demonstrating that using BNTES organosilane up to 10% in the synthesis medium did not prevent the surfactant-templated growth of the regular mesostructure.

**Figure 5:**
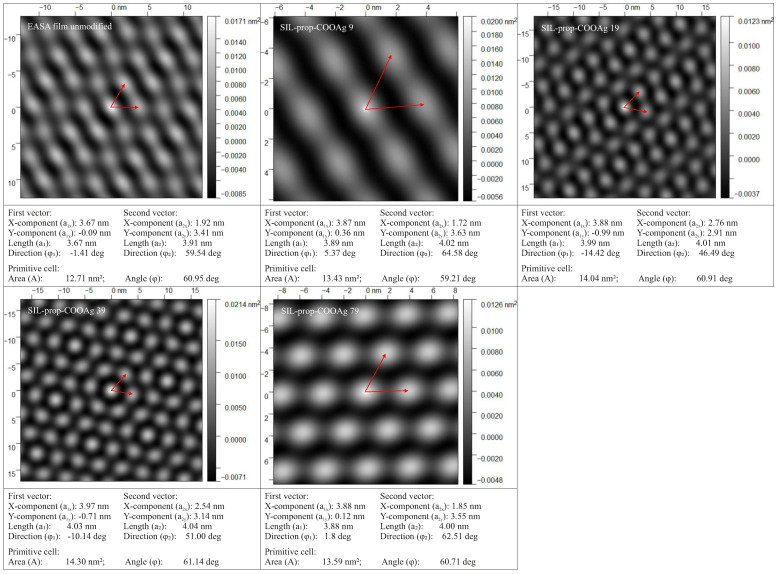
ACF processing of TEM images of investigated materials juxtaposed with unmodified thin silica film. Samples with marked elemental cell vectors, along with pore distribution parameters.

We processed the TEM images using the 2D autocorrelation function (ACF) [47] to confirm the regular 2D hexagonal pore arrangement and find exact pore distribution parameters. The results obtained for the investigated samples, along with the pore distribution parameters, are shown in Figure 5. The obtained results confirmed that the pores are distributed hexagonally 2D, and the distance between the pores centers is about 4 nm. No significant differences between functionalized samples and non-modified films can be noticed. As can be clearly seen, the obtained results confirmed that the pores are distributed hexagonally 2D (the angle between elemental cell vectors is close to 60°). Considering the fact that the samples were placed perpendicular to the electron beam direction, a regular distribution seems to be unambiguously confirmed.

Dealing with the presence of silver species in the mesoporous films, they were not likely to be detected by the sensitive X-ray photoelectron spectroscopy (XPS) method, most probably because they are mainly located deeper in the mesopores, and silver-containing specimens have a low photoelectric cross-section and are very hard to detect in small amounts under XPS measurements [48,49]. EDS elemental analysis, in turn, scans relatively deep [50]. Taking into consideration the thickness of investigated thin films—no more than 100 nm, the EDS signal is dominated by elements creating substrate: silicon, tin, oxygen, and fluor. The signal originating from silver practically cannot be distinguished from the background, and quantitative analysis of the silver content was impossible based on EDX measurement. Similarly, the vibrational analysis also was not able to detect efficiently silver content in the sample, since vibrations originating from Ag-O groups (around 240 cm${}^{-1}$) are overlapped by very intense FTO bands [51,52]. In addition, in this case, the crucial part of spectra is dominated by the signal from the substrate. An alternative technique that is likely to detect even trace amounts of the metal ions in such samples is differential pulse anodic stripping voltammetry [29]. In doing so, the metal ions are first electrochemically reduced and then quantitatively analyzed by anodic stripping. Here we must remark that it is virtually the only method for relative quantification of the amount of the silver ions in our SIL-prop-COOAg thin films.

**Figure 6:**
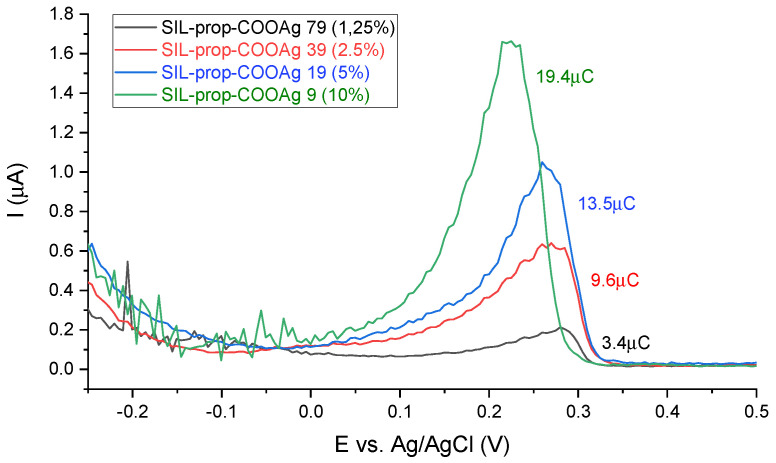
Differential pulse anodic stripping voltammetry (DPASV) curves recorded after 1 min electrolysis at −0.25 V in 0.1 M NaNO${}_{3}$ electrolyte solution using silver-functionalized samples deposited on FTO-covered glass: vertically aligned porous silica thin films containing various contents of silver ions anchored via propyl-carbonate units. Conditions of experiment: pulse height of 2.5 mV, pulse width of 100 ms, step height of 5 mV and step time of 500 ms.

For the reason quoted above, we applied DPASV for the verification of the synthesis procedure by measuring the relative amount of the silver in prepared samples that were thus applied as working electrodes in a pure electrolyte solution. Since the working solution contained no silver ions, the DPASV signals are only related to the ones present in the functionalized films on the surface of the working electrode. Typical DPASV results for the samples with different numbers of anchored functional groups are depicted in Figure 6. After electrolysis at 0.25 V for 1 min (leading to Ag${}^{\left(I\right)}$ reduction into Ag${}^{\left(0\right)}$ onto the electrode surface), one can see the presence of stripping peaks for all samples at approximately 0.2–0.3V corresponding to the reoxidation of silver deposits, the surface of which being proportional to the amount of silver on the electrode [53].

**Figure 7:**
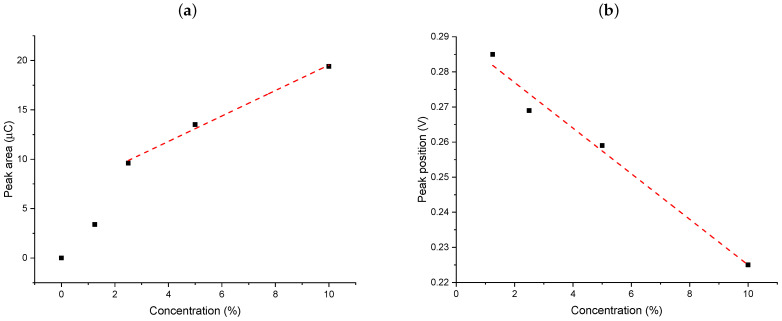
The dependencies of peaks surface areas (**a**) and peaks potentials (**b**) on the functionalization level (mol % of BNTES in the synthesis medium, corresponding to assumed concentration of the silver in the samples). Data originates from experiments of Figure 6.

Taking into consideration that this peak is absent for the non-activated films and the variation of peak areas plotted versus the functionalization level (Figure 7a), this confirms the presence of increasing contents of silver-containing groups in the films. The variation is linear for samples from 2.5% to 10% functionalization, but the response of the 1.25% sample seems to be underestimated, which can be due to less electrochemically accessible silver ions due to their dilution in the materials (-prop-COOAg moieties farther from each other). A similar restriction of charge transfer has been reported for redox-active groups covalently attached to such oriented mesoporous films for which the electron hopping between adjacent groups became dramatically hindered at functionalization levels below 2.5% [39]. Finally, the asymmetric peaks profiles indicate rather slow anodic stripping, with a fast drop in currents after having reoxidized all Ag${}^{\left(0\right)}$ formed during the electrolysis performed 0.25 V for 1 min prior to DPASV.

The peak positions are also informative as they vary with the concentration of functional groups in the films, decreasing by ca. 60 mV when passing from SIL-prop-COOAg 79 to SIL-prop-COOAg 9 samples (Figure 7b). This was unexpected as stripping peak potentials are usually evolving in the opposite direction when increasing the number of metal species to be stripped at chemically modified electrodes (due to the longer time needed to oxidize larger metal deposits) [53,54]. In the present case, the oxidation of silver during the anodic stripping is thus facilitated when occurring at film electrodes bearing more concentrated carboxylate groups, which can be explained by their stabilizing effect in anchoring the Ag${}^{+}$ species generated by DPASV, confirming once more the successful functionalization of the films.

**Figure 8:**
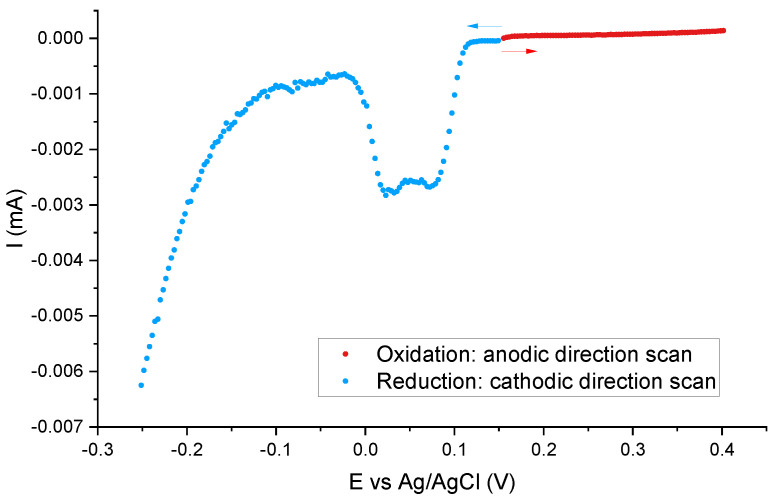
The test of the redox state of silver-containing molecules: linear potential scans of the SIL-prop-COOAg 9 samples in 0.1 M NaNO${}_{3}$ electrolyte solution. For anodic and cathodic directions, separate samples were used. Potential scans were carried out from the neutral (zero-current) potential, equals 0.15 V.

The most probable configuration of silver is an ionic form Ag${}^{+}$. However, in order to confirm this thesis, we performed an additional experiment. To evaluate the redox state of the silver-containing molecules, one can scan potentials in a linear way from neutral (the zero-current potential, to avoid any transformation) towards negative values and positive ones (with the use of separate samples). When the signal occurs during scanning potentials towards the cathodic direction, it would mean that the material contains some Ag${}^{+}$ moieties. Scanning towards the anodic direction, in turn, can reveal Ag${}^{0}$ when the signal detects. The results of such an experiment can be seen in Figure 8. To increase the resolution, we did measurements for the sample containing the highest number of functional units: SIL-prop-COOAg 9.

As it can be clearly seen, the reduction peak is clearly seen, while we cannot observe any oxidation one. This proves that our samples contain ionic silver Ag${}^{+}$.

**Figure 9:**
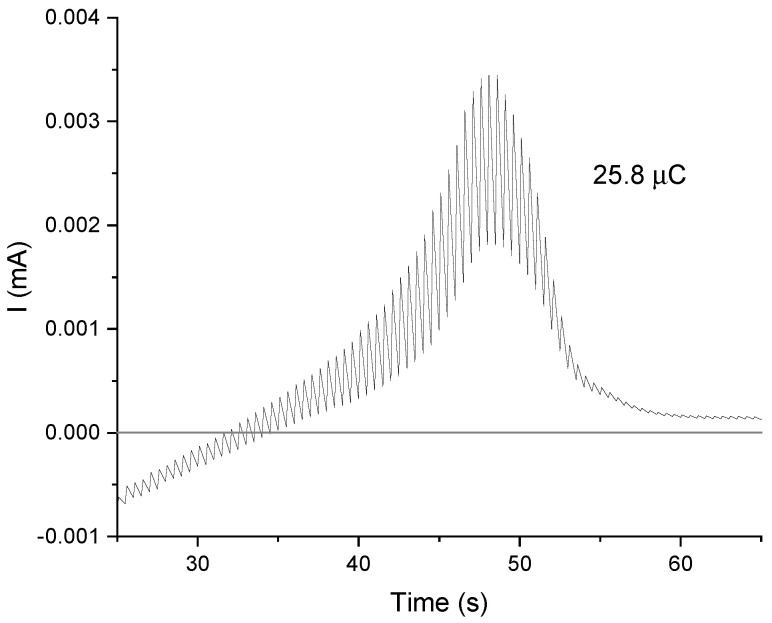
Dependence of current on time during the DPASV measurement for the sample of SIL-prop-COOAg 9 (containing 10% of functional groups) with depicted peak area corresponding to the charge transferred during the electrode reaction.

Plotting the current values versus time for the oxidation reaction during DPASV measurement (Figure 9), one can obtain the charge, *Q*, corresponding to the silver oxidation process by integrating the surface area under the curve. Knowing this value, it is possible to estimate approximately the number of electrochemically accessible silver ions in one pore. Indeed, this charge is directly related to the number of moles of silver involved in the reaction by the Faraday law Q=n×m/M×F where *n* is the number of exchanged electrons (1 in this case), *F* is the Faraday constant (96,485 C× mol${}^{-1}$) and m/M (mass on molar mass) corresponds to the number of moles). In the particular case of the sample with the highest concentration of silver ions inside pores—sample SIL-prop-COOAg 9—the calculated charge of 25.8 μC would lead to ions number ($N_{ions}$) that can be calculated as follows (by taking into account the Avogadro number *N* (6.02 × 10${}^{23}$ mol${}^{-1}$):

(1) $$N_{ions}=\frac{Q_{el.proc}}{F}\times N=\frac{25.8\times 10^{-6}C}{96485\;C\times {\mathrm{mol}}^{-1}}\times 6.02\times 10^{23}\;{\mathrm{mol}}^{-1}=16.1\times 10^{13},$$

where $Q_{el.proc}$ is the charge corresponding to the process.

Dividing this number into the number of pores on the electrode surface, we will obtain the minimal approximate number of ions in one pore. The pore density in such kind of thin films is 7.5 × 10${}^{12}$ pores per 1 cm^2^ [23].

The surface area of the working electrode was 0.49 cm${}^{2}$, thus the pores number in this area ($N_{pores}$) was 3.7 × 10${}^{12}$. This allows for the calculation the number of ions inside single channel according the following formula:

(2) $$N_{ions/channel}=\frac{N_{ions}}{N_{pores}}=\frac{16.1\times 10^{13}}{3.7\times 10^{12}}=43.5ions/channel$$

It is worth noticing that this number of ions in a pore is a rough value that could be underestimated as one cannot be sure that all silver species are electrochemically accessible. Nevertheless, the number obtained here indicates that multiple ions can be placed in one pore, and their number can be tuned.

On the other hand, assuming a pore length equal to 100 nm, the volume of a single pore will be 314 nm${}^{3}$. This means that one can have 43.5/3.14 × 10${}^{-19}$ = 13.8 × 10${}^{19}$ ions per cm${}^{3}$, corresponding to 2.3 × 10${}^{-4}$ moles per cm${}^{3}$, which is in the order of magnitude of organic group contents that one can have in MCM-41 materials obtained by the co-condensation route [55]. On the basis of these considerations, the obtained value of 40–45 ions per channel would indicate quite a satisfactory filling.

## 4. Conclusions

In conclusion, we have reported a synthesis technique to fabricate the vertically aligned mesoporous silica thin films functionalized by silver ions inside pores, based on the electro-assisted co-condensation method. We assumed that the silver ions could be anchored inside pores via propyl-carboxyl units. In our opinion, such an approach could allow for controlling the number of immobilized ions, contrary to the grafting procedure. To check this, we prepared materials with various content of silver-containing groups. The resulting thin layers presented a very high level of pores’ ordering. Therefore, the organic groups and attached metal ions did not disrupt the process of vertically aligned porous thin film growth.

The obtained material has turned out to be very difficult for investigation with regard to molecular structure, and spectroscopic methods were hard to apply. However, we have shown that the differential pulse anodic stripping voltammetry is an ideal method for the investigation of such materials for the reason of very high sensitivity. Based on electrochemical measurements, we confirmed the presence of the metal ions in the structure of the film and showed the direct dependency between the assumed number of anchoring units and the quantity of attached silver. On this basis, we can conclude about the efficiency of the presented method.

What is more, we calculated the number of silver ions occupying a single pore for the highest concentration of functional units. Considering the obtained value, we proved that the multiple ions could be placed in a single pore, and this number can be tuned during synthesis. We are convinced that the presented method can be easily generalized for other metals and anchoring units.

## Data Availability

Not applicable.

## Abbreviations

The following abbreviations are used in this manuscript:

EASA	electro-assisted self-assembly
FTO	Fluoride doped Tin Oxide
CTAB	cetyltrimethylammonium bromide
TEOS	tetraethyl orthosilicate)
BNTES	butyronitrile triethoxysilane
TMSCl	trimethylsilyl chloride
